# Oral Health Practices Among Indigenous Mothers and Young Children (0–36 Months) in Northwest Territories, Canada

**DOI:** 10.1155/ijod/7094004

**Published:** 2025-12-01

**Authors:** Fariba Kolahdooz, Moutasem Zakkar, Se Lim Jang, Claire Manning, Tyler Verhaeghe, Cindy Roache, André Corriveau, Debbie DeLancey, Adrian Wagg, Marie Tarrant, Sangita Sharma

**Affiliations:** ^1^Indigenous and Global Health Research Group, Department of Medicine, Faculty of Medicine and Dentistry, College of Health Science, University of Alberta, 1-126 Li Ka Shing Centre for Health Research Innovation, 8602 112 Street, Edmonton, Alberta T6G 2E1, Canada; ^2^Mike Petryk School of Dentistry, Faculty of Medicine and Dentistry, College of Health Sciences, University of Alberta, Dianne and Irving Kipnes Health Research Institute, 5th Floor, 11405 87 Avenue, Edmonton, Alberta T6G 1C9, Canada; ^3^Independent Public Health Consultant, Yellowknife, Northwest Territories, Canada; ^4^Aurora College, 5004 54 St, Yellowknife, Northwest Territories X1A 2R6, Canada; ^5^Division of Geriatric Medicine, Department of Medicine, Faculty of Medicine and Dentistry, College of Health Sciences, University of Alberta, 1-198 Clinical Sciences Building, 11350 83 Avenue, Edmonton, Alberta T6G 2B7, Canada; ^6^Faculty of Health and Social Development, University of British Columbia Okanagan Campus, 1147 Research Road, Kelowna, British Columbia V1V 1V7, Canada

**Keywords:** early childhood caries, Indigenous, maternal, oral health

## Abstract

**Objectives:**

Early oral hygiene and care help prevent early childhood caries (ECC). As remote Indigenous communities in Canada have limited access to dental care, this project explores Indigenous women's oral hygiene practices during and after pregnancy and in regard to children (0–36 months) within three communities in Northwest Territories (NWT), Canada.

**Methods:**

A mixed-methods study design was employed. Self-identifying Indigenous women who were pregnant or had given birth within the 3 years preceding data collection (2019) were invited to complete a semi-structured, interviewer-administered questionnaire.

**Results:**

156 Indigenous women participated. Of participants who gave birth in the last 3 years (*n* = 145), 78.8% started brushing infants' teeth/gums. Of participants whose infants had teeth (*n* = 114), 27.9% had taken the infant to a dentist. Factors impacting infant oral hygiene practices included personal experiences, access to supplies, and health literacy. Among pregnant participants (*n* = 28), 38.5% had visited a dentist during pregnancy. Among participants who had given birth within 3 years (*n* = 145), 67.9% had since seen a dentist. Factors impacting dental care utilization during pregnancy included service availability and safety concerns. After giving birth, factors included service availability and competing priorities.

**Conclusions:**

Within Indigenous communities in NWT, inaccessible oral health care, information, and supplies made it challenging for mothers to meet oral health recommendations during and after pregnancy and in regard to young children. The findings of this project suggest the need for long-term, collaborative strategies focused on increasing the availability and accessibility of dental care professionals and services within communities and improving oral health literacy via culturally appropriate and collaboratively developed informational initiatives.

## 1. Introduction

In Canada, provincial public health insurance programs do not cover dental care, and the resulting financial burden can be a barrier to accessing dental care [1]. The Government of Canada is currently implementing the Canadian Dental Care Plan, aiming to provide dental coverage to residents of Canada under a certain income threshold and without dental insurance [2]; however, its efficacy is currently unknown. Many Indigenous community members in Canada are additionally eligible for dental coverage through Non-Insured Health Benefits (NIHB) [3, 4], and the Children's Oral Health Initiative (COHI) provides preventative dental care to Indigenous children (0–7 years old) [5]. However, in the Northern territories of Canada (Northwest Territories (NWT), Nunavut, and Yukon), where most individuals are Indigenous, utilization rates of dental care services remain low [4]. In addition to affordability, access to dental care services within Indigenous communities is disproportionately affected by several systemic determinants beyond the individual level, such as service availability, accessibility, accommodation, and government policies [6].

Available literature on access to and the provision of dental care services for Indigenous communities is limited to communities in the southern regions of Canada [6], where services, while potentially distant, are available. In the Northern territories of Canada, dental care service provision is more challenging. NWT is located north of the 60th parallel, and, among the Canadian provinces and territories, NWT has the second-highest proportion of Indigenous residents (50.0%), many of whom continue to speak an Indigenous language (33.2%) and practice Indigenous traditions (36.3%), such as hunting and fishing [7, 8]. Within NWT, many communities are remote, few have all-season road access, several are fly-in only, and only three of the 33 dispersed communities have private dental clinics [9]. Five communities have a dental hygienist or dental therapist and offer prenatal and early childhood oral health programs. Residents living in other communities must rely on infrequent visits from dental care professionals or must travel to surrounding communities to access dental care [9–11], medical travel itself being challenging and a potentially limiting factor to accessing care [12]. This limited accessibility is reflected in the disproportionately higher risk for dental disease reported within Indigenous communities compared to non-Indigenous communities [5]. The rate of untreated dental caries in Indigenous adults has been reported at 34.4%, whereas the rate in non-Indigenous adults was 19.3% [13]. Critically, limited access to dental care extends and is detrimental to the oral health of Indigenous children [14].

When a child presents with one or more decayed (due to a non-cavitated or cavitated lesion), missing (due to caries), or filled primary teeth, it is referred to as early childhood caries (ECC) [15, 16]. The primary etiology of ECC involves ingested sugars creating an acid that demineralizes tooth enamel and penetrates into the tooth [17]. ECC is therefore associated with a high-sugar diet, as well as insufficient vitamin D and calcium intake [18], high-sugar diets having become increasingly common and thus impactful among Indigenous children [19, 20]. Parents' socioeconomic status (SES) has also been associated with ECC [21]. SES affects an individual's health behaviors and the relative affordability of dental care [21]. Children of parents with low SES may be more likely to have ECC and poorer oral health than children of parents with high SES [22–24]. Health literacy, which denotes “the degree to which individuals can obtain, process, and understand the basic health information and services needed to make appropriate health decisions” [25], also affects an individual's exposure to risk factors that affect health, including oral health [25–28], with low education level being associated with a lower level of oral health literacy [29].

About half of children aged 6–11 years in Canada experience ECC [13]. The detrimental effects of poor dental health in children are well documented. Disease in primary teeth affects children's nutritional attainment and communication abilities [30] and can lead to challenges throughout life, including growth, developmental, educational [31], social, and financial challenges [13, 15–17, 32–34]. ECC can also increase the risk of respiratory and/or cardiovascular diseases in adulthood [34]. Critically, Indigenous children in the Northern territories in Canada have higher incidence rates of ECC [31] than non-Indigenous children in provincial urban communities [13, 14, 20, 35, 36].

Strategies to prevent ECC include checking and brushing infants' teeth and gums, avoiding sugary liquids on pacifiers and in bottles, and not allowing infants to sleep with a bottle [37], mothers chewing xylitol gum to prevent the transmission of mutans streptococci to infants [38], using fluoridated toothpaste and water fluoridation [14, 15]. Findings about children chewing xylitol gum are inconclusive; however, some suggest benefits among children with signs of tooth decay [39–41]. Early involvement of caregivers and healthcare professionals is also critical [14]. It is recommended to initiate oral health maintenance prior to the eruption of the first tooth, which usually occurs around 6 months of age [37, 42, 43]; visiting the dentist for the first time at a later age increases the risk of ECC [44].

Women maintaining oral health during pregnancy can also benefit the health of children. Pregnant women are at an increased risk for gingivitis (due to hormonal changes), tooth decay and erosion (due to morning sickness), and periodontitis [45]. Periodontitis during pregnancy may result in low birth weight, preeclampsia, and preterm delivery [3, 46]. While alternative interventions, such as xylitol gum, may help prevent periodontitis and related birth outcomes [47], pregnant women are recommended to visit a dentist during the first trimester for teeth cleaning and an oral health exam; however, the rates of women accessing dental care during pregnancy are not well known [48].

Very limited literature on the oral health of mothers, infants, and young children in NWT is available. Thus, this article explores Indigenous women's oral hygiene practices during and after pregnancy and in regard to infants (0–12 months) and young children (12–36 months), as well as the factors affecting the utilization of such practices. In this article, dental care refers to the care received when visiting a dentist or other dental care professionals, and infant oral hygiene refers to practices a mother implements to protect and preserve her child's oral health.

## 2. Materials and Methods

### 2.1. Setting

This project is part of the Maternal and Infant Health Project, which explored the availability of, access to, and utilization of healthcare services among Indigenous women, including dental care. Detailed materials and methods have been published [49]. This project took place in three NWT communities, previously described, where 23%–90% of community members are Indigenous [50–52]: Community A is a remote community which relies on visiting dentists for dental care [52, 53]; Community B is a semi-remote administrative center with one dental clinic [51, 54]; and Community C is a major city with multiple dental clinics [50, 54].

### 2.2. Project Design

This study used a cross-sectional mixed-methods design, integrating quantitative and qualitative data collection approaches to reflect the lived experiences of Indigenous mothers and the contextual factors shaping oral health practices. Purposive recruitment methods, which were suggested by the Community Advisory Board (CAB), were utilized to recruit self-identifying Indigenous women who were currently pregnant or who had given birth within the past 3 years. The response rate was 90%. Non-participation was primarily due to limited availability and lack of interest.

### 2.3. Data Collection

Utilizing an interviewer-administered semistructured questionnaire, data were collected between October 28 and November 9, 2019, on pregnancy history, health status, oral health, sources of health information and support, and the healthcare services accessed during pregnancy, birth, and the postnatal period.

Oral health practices were explored using closed-ended questions (e.g., “Have you seen a dentist during your pregnancy?” and “Have you started brushing your baby's gums or teeth?”). Participants were also invited to share stories relating to oral health maintenance and practices during and after pregnancy. The questionnaire was developed in collaboration with the CAB and pilot-tested for cultural relevance and clarity.

Interviews were conducted by local Indigenous research assistants who were recruited from each participating community. Prior to data collection, all interviewers completed a multiday training program led by the study team, which included instruction on research ethics, culturally safe interviewing techniques, and the use of the semi-structured questionnaire. Mock interviews were conducted to ensure confidence and consistency. To maintain fidelity, the study team was present on-site during data collection to provide real-time support and oversight. Interviews were conducted at a private venue of the participants' choice and lasted ~30 min. While translation into local languages was available upon request, all participants chose to complete the interviews in English. With participant permission, responses were audio recorded, transcribed verbatim, and entered into REDCap report forms. All participants received a $25 gift card as an honorarium to a local grocery store.

The interview process, described in full previously [49], was undertaken with the understanding and written consent of each participant and in full accordance with ethical principles, including the World Medical Association Declaration of Helsinki (version 2008), and territorial and federal laws. Ethical approval for this project was granted by the corresponding author's institutional review board, the Aurora Research Institute in NWT approved the research license, and a research agreement was signed with the Department of Health and Social Services, Government of NWT.

### 2.4. Data Analysis

Quantitative data analyses were performed using SAS statistical software, version 9.4 (SAS Institute Inc., 2013). Fisher's exact and chi-square tests were used to examine whether infant and maternal oral health practices differed by community. All analyses were conducted using complete cases. Responses marked as “I don't know” were included in the analysis as valid categorical responses. Only unanswered questions were treated as missing data and excluded from summary statistics.

For open-ended responses, content analysis was performed utilizing NVivo-Pro in an interactive process previously described [49]. Briefly, responses were transcribed and imported into NVivo-Pro software. An inductive coding approach was used to identify recurring ideas and patterns. Two researchers independently reviewed the data and developed thematic categories through iterative coding and discussion. Themes were refined collaboratively and grouped into broader domains to complement the quantitative findings. When reporting these themes, and in consultation with Indigenous partners and the CAB, it was collectively decided not to include participant identifiers alongside quotations. This decision reflects a commitment to cultural sensitivity and honors the lived experiences of Indigenous community members. Historically, Indigenous individuals were often referred to by numbers rather than names in institutional systems, a dehumanizing practice tied to Canada's colonial legacy and the resulting intergenerational trauma. Details such as gender or age were also not reported, as some participating communities have fewer than 300 members. Including such descriptive information could unintentionally make individuals identifiable through the quotations.

## 3. Results

In total, 156 Indigenous women participated. Participants' ages ranged from 17 to 47 years old, with one participant refusing to disclose her age. Mean age, level of education, and work status are presented in Table 1.

### 3.1. Infant Oral Hygiene and Healthcare

At the time of data collection, 78.8% of participants who had given birth within the past 3 years had started brushing infants' teeth/gums, 63.0% of whom reported that teeth and gum brushing began within or before the CDA-recommended timeframe of 6 months of age. Among participants whose baby's first tooth had appeared, 27.9% had taken the infant to a dentist, with 38.7% of participants doing so for the first time after the child reached 19 months of age (Table 2). No statistically significant associations were found between the communities and any of the infant or maternal oral health variables tested (*p* > 0.05).

Participants described several factors influencing the initiation and maintenance of infant oral hygiene practices, including personal experiences, health literacy (i.e., knowledge and understanding of health promotional materials and awareness of infant oral hygiene practices), and the availability of adequate oral health supplies.

#### 3.1.1. Personal Experiences

Several participants described how personal experiences informed the perceived importance of and thus initiation of infant oral hygiene practices:

“I think you have to. [I] see so many kids with no front teeth. [I] have a lot of cousins that grow up in environments where oral hygiene isn't practiced. Just important to me.”

“I didn't do it with my first, and she ended up getting thrush, so lesson learned.”

#### 3.1.2. Health Literacy

Some participants expressed awareness of infant oral hygiene practices, while others did not:

“I know it is very beneficial. Oral health is one of the key things to prevent long term health issues in the long run.”

“Nobody told me it.”

The timing of teeth eruptions and awareness of specific practices, such as gum brushing, impacted the initiation of infant oral hygiene practices:

“No teeth coming in yet.”

“Too early—but I was going to start rubbing her mouth so it can be clean.”

Participants reported that health promotion materials posted in community organizations and healthcare settings informed the initiation of infant oral hygiene practices:

“[I] had a pamphlet, and it said that it was important, so I did it.”

Advice from healthcare professionals further raised awareness of infant oral hygiene needs:

“My health provider told me that the breast milk would stay on her tongue and gums, causing a little rot.”

#### 3.1.3. Access to Supplies

Participants reported utilizing various tools to brush infants' teeth and gums, including cloths, finger brushes, and brushes:

“Just with a cloth. To clean her mouth out.”

“When he was a baby, I was using a cloth on his gums.”

“I wiped gums and brushed when he had teeth.”

While most participants had initiated gum brushing, a few had not, with some participants citing limited access to brushing supplies:

“We were talking about the little gummy toothbrush; it will happen soon. With [an older child's name], it was given to me, but I guess they don't give them out anymore.”

“Because [I] didn't get a gum brush.”

### 3.2. Mother's Dental Care

Of the participants who were pregnant at the time of the project, 38.5% reported visiting a dentist during pregnancy (Table 3). Three main factors impacted participants' utilization of dental care during pregnancy: service availability, safety concerns, and competing priorities.

#### 3.2.1. Service Availability

Several participants who had not seen a dentist during pregnancy cited the limited availability of dental services in NWT:

“When I first called them [the dental clinic], I was in my first trimester, and they could not help. Called in third trimester, [they] said they couldn't come because they only come twice per year to my community.”

“It is really hard to get dentist appointments. By the time you call a dentist to make an appointment they're booked up. They only come every four months.”

“I need to make an appointment. The dental wait is so long here.”

“We don't have one in [Community B] anymore.”

#### 3.2.2. Safety Concerns

Some participants also expressed concern about the safety of visiting a dentist while pregnant:

“Are you allowed to get dental work done… [while pregnant]? Like how far along [can you get dental work done]?”

“Not safe during pregnancy.”

Participants who had given birth within the 3 years prior to data collection (*n* = 145), 67.9% of whom had seen a dentist since giving birth, reported an additional barrier to accessing dental care: competing priorities.

#### 3.2.3. Competing Priorities

Competing priorities related to caring for children impacted participants' ability to access dental services:

“I don't spend a lot of time thinking about my health as it all goes to the kids.”

“I don't have the time as I'm co-parenting with her dad and I barely have time. And when I have free time I'm too tired.”

## 4. Discussion

This project explored maternal oral hygiene practices during pregnancy, after pregnancy, and in regard to infants and young children within three NWT communities. While participants made significant efforts to initiate and maintain infant oral hygiene practices, such efforts were impacted by many factors, including the mother's exposure to and understanding of oral health promotional materials, access to oral hygiene supplies, and ability to allocate time for infant oral hygiene. Further evaluating oral health literacy among mothers, as well as the current accessibility of oral hygiene information and tools in NWT, could inform future oral health initiatives. Ensuring that informational resources and initiatives are culturally relevant and developed in collaboration with communities may help improve the effectiveness of oral health promotion [55, 56]. Data on the incidence of dental cavities in children in Northern Canada is scarce; the latest nationally organized survey on oral health in Northern Canada was conducted in early 2000s [57]. Studies found that 66% of preschool-aged children in one region of NWT had dental caries, with an average of five affected teeth in 2004–2005 [36], and 69% of parents of preschool-aged children across Nunavut reported dental caries in 2007 [58]. Mothers' understanding of the importance of promoting children's oral health and awareness of proper oral health behavior is critical in improving children's oral health behaviors and reducing the risks for oral caries among children [59, 60]; therefore, improving children's oral health must involve mothers and other caregivers. An urgent need for prevention-focused dental health strategies for children in NWT has been identified [61], and this study provides long overdue evidence to inform practices.

Compared to the proportion of participants initiating infant oral hygiene practices, the proportion accessing infant dental care was lower. While comparable data on initial dental visit age within the Northern territories is not available, children in the Northern territories do experience more challenges accessing dental care than children in other parts of Canada due, in part, to geographic obstacles and dental care professional shortages [5, 62]. As participants in this project discussed, recruiting and retaining dentists to work in and travel to remote Indigenous communities in NWT is a challenge [14, 63]. The availability of dental therapists, who travel to provide dental health programs and services in NWT communities without dental clinics, is also limited. Within Canada, it is approximated there are fewer than 200 dental therapists, and currently, only one program exists to train new dental therapists [64–66].

The proportion of participants who visited a dentist during pregnancy was lower than what has been reported in comparable studies conducted in Canada. In British Columbia, a study of non-Indigenous pregnant women from diverse ethnic backgrounds found that 50% of participants had not visited a dental care professional in the past year [48]. Further, a study of pregnant Indigenous women in Manitoba and Ontario found that 66.5% had seen a dentist in the past 12 months [62]. Although study designs and population characteristics may vary considerably, the results of this project suggest that regional and demographic disparities regarding dental care exist within Canada and require further exploration.

In addition to the limited availability of dental care, concerns about the safety of visiting a dentist during pregnancy impacted participants' utilization of dental care. Similar beliefs related to the safety and importance of dental care during pregnancy were reported in other contexts [67, 68]. As well, uncertainty among dental care professionals regarding the safety of providing care to pregnant patients may additionally reduce access to dental care [69, 70]. Future projects could formally evaluate knowledge and beliefs surrounding the utilization of dental care during pregnancy within Indigenous communities, among both pregnant women and dental care providers, to inform future initiatives.

The proportion of participants who had visited a dentist since giving birth was higher than the proportion of pregnant participants but still lower than the average for dental visits among women across Canada's provinces [1]. While population- and geographic-specific information on dental visits among new mothers in NWT does not exist outside of the national figures, research in 2018 indicated that access to dental care within some NWT communities had increased in recent years [71]. However, factors such as the COVID-19 pandemic may have disrupted the availability of traveling dental care professionals and the provision of dental care in NWT communities [72, 73]. The impact of such disruptions on the already limited availability of dental care professionals and services within remote Indigenous communities warrants further investigation.

In Canada, several programs are working to support oral health in remote communities. Implemented in 2004, COHI has successfully helped deliver preventative oral health services to 320 Indigenous communities across Canada [5] and improve caregiver knowledge of infant oral health [55]. However, in remote communities, the implementation of this initiative may have been restricted by a lack of dental care professionals [5]. The Government of NWT also released the Oral Health Action Plan in 2018 to improve children's oral health [61]. Pregnant women in five of the 33 NWT communities can also currently access the Prenatal Oral Health Program, which provides free oral health education and screening to pregnant women, with a parallel program available for children 0–4 years old. The outcomes of this intervention have yet to be studied [74], but if effective, the intervention could be implemented in more communities.

This project adds to the currently limited information exploring infant and maternal oral health and hygiene within remote Indigenous communities in NWT. One limitation of this study is that data on caries prevalence among infants and mothers were not collected, preventing assessment of its relationship with oral health practices. As well, this project did not differentiate between the types of dental care professional participants could access or between the methods utilized to access care (private dental clinic, medical travel, etc.). Due to the utilization of purposive recruitment methods, the results cannot be generalized to all Indigenous communities without considering the different contexts. While Indigenous communities in Canada share historical traumas and experiences, communities also have distinctive traditions and values that require consideration, and which further affect generalizability. However, the recruitment methods utilized were deemed the best approach to recruit as many eligible participants as possible and gather as much information as possible. Some variables may have missing data due to recall limitations. Although an “I don't know” option was provided and included in the analysis, unanswered questions may reflect participants' difficulty recalling specific timeframes or events, a common challenge in retrospective self-report surveys.

## 5. Conclusions

Within Indigenous communities in NWT, there are challenges in meeting oral health recommendations for mothers during and after pregnancy and for children under the age of three. For young children and infants, oral health may be impacted by mothers having limited access to oral health information, tools, and care. For both mothers and young children, the limited availability of dental care professionals may impact oral health. Long-term improvement regarding the oral health of young children and mothers will require collaboration with Indigenous community members and leaders, healthcare professionals, and policymakers. Intervention strategies should focus on improving oral health literacy through culturally and language appropriate promotional materials, as well as increasing the availability and accessibility of dental care professionals, dental care facilities, and preventive oral health programs within remote Indigenous communities.

## Figures and Tables

**Table 1:** Demographic characteristics of Indigenous mothers in three communities in the Northwest Territories (*n* = 156).

Variable	Community			
	A	B	C	Total
Participants, *n* (%)	24 (15.4)	68 (43.6)	64 (41.0)	156 (100)
Mean, Age (SD)	32.0 (5.8)	29.3 (6)	29.1 (6.0)	29.7 (6.0)

	***n* (%)**	***n* (%)**	***n* (%)**	***n* (%)**

Pregnant^ac^				
Yes	3 (12.5)	13 (19.1)	12 (18.8)	28 (18.0)
No	20 (83.3)	54 (79.4)	52 (81.3)	126 (80.8)
Gave birth in last 3 years				
Yes	22 (91.7)	64 (94.1)	59 (92.2)	145 (93.0)
No	2 (8.3)	4 (5.9)	5 (7.8)	11 (7.1)
Age categories (years)^c^				
<25	2 (8.7)	14 (20.6)	15 (23.4)	31 (20)
25–35	14 (60.9)	45 (66.2)	39 (60.9)	98 (63.2)
>35	7 (30.4)	9 (13.2)	10 (15.6)	26 (16.8)
Ethnicity^ac^				
First Nations	24 (100)	17 (25.4)	41 (66.1)	82 (53.6)
Inuit	—	43 (64.2)	7 (11.3)	50 (32.7)
Inuit and First Nations	—	5 (7.5)	3 (4.8)	8 (5.2)
Métis	—	—	11 (17.7)	11 (7.2)
Education^a^				
Less than or some high school	17 (70.8)	29 (42.7)	21 (32.8)	67 (43.0)
High school diploma or equivalent	3 (12.5)	12 (17.7)	14 (21.9)	29 (18.6)
Postsecondary education	4 (16.7)	27 (39.7)	28 (43.8)	59 (37.8)
Employment status^c^				
Full time	5 (20.8)	17 (25.4)	21 (32.8)	43 (27.7)
Part time	5 (20.8)	9 (13.4)	2 (3.1)	16 (10.3)
Maternity	2 (8.3)	9 (13.4)	9 (14.1)	20 (12.9)
Student	—	6 (9.0)	3 (4.7)	9 (5.8)
Not working^b^	12 (50)	26 (38.8)	29 (45.3)	67 (43.2)

^a^Levels of five or fewer observations were omitted.

^b^Not working includes the following response options: not working and looking for a job; not working and not looking for a job, and unable to work.

^c^Missing data were omitted from all analyses.

**Table 2:** Oral hygiene and oral healthcare practices for mothers and infants (*n* = 145).

Variable	Community			
	A	B	C	Total
	*n* (%)	*n* (%)	*n* (%)	*n* (%)
Mother started brushing baby's gums or teeth^ae^				
Yes	13 (68.4)	53 (84.1)	42 (76.4)	108 (78.8)
No	6 (31.6)	10 (15.9)	11 (20)	27 (19.7)
Baby's age (months) when mother started brushing gums/teeth^be^				
<2	—	12 (24)	6 (15)	18 (18)
2–4	3 (30)	10 (20)	5 (12.5)	18 (18)
>4–6	6 (60)	14 (28)	7 (17.5)	27 (27)
>6–8	1 (10)	7 (14)	14 (35)	22 (22)
>8	—	7 (14)	8 (20)	15 (15)
Baby's first tooth had appeared^ae^				
Yes	18 (90)	52 (82.5)	44 (77.2)	114 (81.4)
No	2 (10)	10 (15.9)	12 (21.1)	24 (17.1)
Baby's age (months) when first tooth appeared^ce^				
<6	11 (84.6)	31 (66.0)	26 (61.9)	68 (66.7)
7–12	2 (15.4)	16 (34.0)	16 (38.1)	34 (33.3)
Mother had taken the baby to see a dentist^ace^				
Yes	3 (17.7)	15 (29.4)	13 (30.2)	31 (27.9)
No	14 (82.4)	35 (68.6)	30 (69.8)	79 (71.2)
Baby's age (months) when first saw a dentist^de^				
<6	—	—	1 (7.7)	1 (3.2)
7–12	1 (33.3)	6 (40)	4 (30.8)	11 (35.5)
13–18	1 (33.3)	1 (6.7)	2 (15.4)	4 (12.9)
≥19	1 (33.3)	6 (40)	5 (38.5)	12 (38.7)

^a^Levels of five or fewer observations were omitted.

^b^Asked only to women who had started brushing baby's gums or teeth, *n* = 108.

^c^Asked only to women whose baby's first tooth appeared, *n* = 114.

^d^Asked only to women whose baby's first tooth appeared and took the baby to see a dentist, *n* = 28.

^e^Missing data were omitted from all analyses.

**Table 3:** Indigenous mothers' visits to a dentist during pregnancy or after giving birth (*n* = 145).

Variable	Community			
	A	B	C	Total
	*n* (%)	*n* (%)	*n* (%)	*n* (%)
Dentist visit during pregnancy^ac^				
Yes	2 (66.7)	5 (41.7)	3 (27.3)	10 (38.5)
No	1 (33.3)	7 (58.3)	8 (72.7)	16 (61.5)
Dentist visit since giving birth^bc^				
Yes	15 (71.4)	41 (67.2)	39 (67.2)	95 (67.9)
No	6 (28.6)	20 (32.8)	19 (32.8)	45 (32.1)

^a^Women who were pregnant at the time of the project, *n* = 28.

^b^Women had given birth in last 3 years, *n* = 145.

^c^Missing data were omitted from all analyses.

## Data Availability

All data supporting the results of this study can be found in the manuscript. De-identified data are owned by the Indigenous communities and may be available upon request with community approval.

## Supporting Information

Supporting Information Completed checklist for the mixed methods appraisal tool.
