# Supplementary material for: Oral Health Practices Among Indigenous Mothers and Young Children (0–36 Months) in Northwest Territories, Canada
Source: Int J Dent. 2025 Dec 1;2025:7094004. doi: 10.1155/ijod/7094004 (PMC12685437; doi:10.1155/ijod/7094004)
Supplement: Supporting Information — Completed checklist for the mixed methods appraisal tool. [file 7094004.f1.docx]

**Mixed Methods Appraisal Tool (MMAT), Version 2018 – Completed Checklist**

Study: Mixed-methods study on oral health practices among Indigenous mothers and young children (0–36 months) in Northwest Territories, Canada

**Part I. Screening questions (for all study types)**

| Criterion | Response | Comments (brief justification) |
| --- | --- | --- |
| S1. Are there clear research questions? | Yes | Objectives and aims clearly articulated in Abstract and Introduction (to examine maternal and infant oral health practices and factors influencing utilization). |
| S2. Do the collected data allow addressing the research questions? | Yes | Mixed-methods design with interviewer-administered semi-structured questionnaire (quantitative items + open-ended responses) provides data aligned with aims. |

**1. Qualitative component**

| Criterion | Response | Comments (brief justification) |
| --- | --- | --- |
| 1.1. Is the qualitative approach appropriate to answer the research question? | Yes | Inductive content analysis of open-ended interview data is suitable to explore underlying factors and experiences. |
| 1.2. Are the qualitative data collection methods adequate to address the research question? | Yes | Semi-structured, interviewer-administered format; local interviewers trained; audio recorded and transcribed; culturally safe and pilot tested. |
| 1.3. Are the findings adequately derived from the data? | Yes | Themes developed iteratively by two researchers with supporting quotations presented in Results. |
| 1.4. Is the interpretation of results sufficiently substantiated by data? | Yes | Interpretations are grounded in participant quotations and aligned with emergent themes. |
| 1.5. Is there coherence between qualitative data sources, collection, analysis and interpretation? | Yes | Data collection (semi-structured interviews), inductive coding by two analysts, and thematic reporting show coherence. |

**2. Quantitative randomized controlled trials (N/A)**

**3. Quantitative non-randomized (N/A)**

**4. Quantitative descriptive component**

| Criterion | Response | Comments (brief justification) |
| --- | --- | --- |
| 4.1. Is the sampling strategy relevant to address the research question? | Yes | Purposive recruitment of Indigenous women who were currently pregnant or had given birth in the last 3 years across three communities is appropriate for the exploratory objectives. |
| 4.2. Is the sample representative of the target population? | Can’t tell | Non-probability purposive sampling was used.  Purposive sampling is not statistically representative; however, given the exploratory nature of the study and the geographic context, including remote communities, some of which are accessible only by plane, random sampling was not feasible. The sampling strategy is appropriate for the study’s aims and setting. |
| 4.3. Are the measurements appropriate? | Yes | Questionnaire developed with community advisory input and pilot tested; items align with constructs (e.g., dental visits, infant brushing). |
| 4.4. Is the risk of nonresponse bias low? | Yes | The study achieved a response rate of approximately 90%, which is considered high for community-based research in remote and Indigenous contexts. Non-participation was primarily attributed to limited availability and lack of interest, rather than systematic differences related to oral health behaviors. Although detailed demographic data on non-respondents were not collected, the combination of a high response rate and transparent reporting of reasons for non-participation suggests that the risk of nonresponse bias is low. |
| 4.5. Is the statistical analysis appropriate to answer the research question? | Yes | Descriptive statistics and Chi-square/Fisher’s exact tests for categorical comparisons by community with complete-case approach are appropriate for cross-sectional descriptive aims. |

**5. Mixed methods component**

| Criterion | Response | Comments (brief justification) |
| --- | --- | --- |
| 5.1. Is there an adequate rationale for using a mixed methods design to address the research question? | Yes | Design explicitly described as exploratory cross-sectional mixed methods to examine practices and explore underlying factors; qualitative findings intended to complement quantitative results. |
| 5.2. Are the different components of the study effectively integrated to answer the research question? | Yes | Qualitative themes were grouped to complement quantitative findings; results and discussion jointly inform interpretation. |
| 5.3. Are the outputs of the integration of qualitative and quantitative components adequately interpreted? | Yes | Discussion integrates quantitative patterns with qualitative explanations to inform implications for programs and policy. |
| 5.4. Are divergences and inconsistencies between quantitative and qualitative results adequately addressed? | Yes | There were no notable divergences or inconsistencies between the quantitative and qualitative findings. Both components were complementary: quantitative data described the prevalence of practices (e.g., infant gum brushing, dental visits), while qualitative themes provided context and explanations (e.g., service availability, safety concerns, competing priorities). The integration of findings in the discussion reflects this alignment. |
| 5.5. Do the different components of the study adhere to the quality criteria of each tradition of the methods involved? | Yes | Qualitative and quantitative descriptive components meet core criteria; limitations (e.g., purposive sampling) are acknowledged. |
